# Efficiency and Patient-Reported Outcome Measures From Clinic to Home: The Human Empowerment Aging and Disability Program for Digital-Health Rehabilitation

**DOI:** 10.3389/fneur.2019.01206

**Published:** 2019-11-19

**Authors:** Sara Isernia, Chiara Pagliari, Johanna Jonsdottir, Carlotta Castiglioni, Patrizia Gindri, Cristina Gramigna, Giovanna Palumbo, Marco Salza, Franco Molteni, Francesca Baglio, HEAD study group

**Affiliations:** ^1^IRCCS Fondazione Don Carlo Gnocchi ONLUS, Milan, Italy; ^2^Fondazione Opera San Camillo Presidio Sanitario San Camillo, Turin, Italy; ^3^Villa Beretta Rehabilitation Center, Ospedale Valduce, Costa Masnaga, Italy

**Keywords:** rehabilitation, technology, telerehabilitation, nervous system diseases, multiple sclerosis, Parkinson's disease, stroke

## Abstract

**Background:** The recent exponential growth of Digital Health (DH) in the healthcare system provides a crucial transformation in healthcare, answering to alarming threats related to the increasing number of Chronic Neurological Diseases (CNDs). New long-term integrated DH-care approaches, including rehabilitation, are warranted to address these concerns.

**Methods:** The Human Empowerment Aging and Disability (HEAD) rehabilitation program, a new long-term integrated care including DH-care system, was evaluated in terms of efficiency and patient-reported outcome measures (PROMs) in 107 CND patients (30 with Parkinson's Disease, PD; 32 with Multiple Sclerosis, MS; 45 with stroke in chronic stage). All participants followed 1-month of HEAD rehabilitation in clinic (ClinicHEAD: 12 sessions, 3/week), then 1:3 patient was consecutively allocated to 3-months telerehabilitation at home (HomeHEAD: 60 sessions, 5/week). Efficiency (i.e., adherence, usability, and acceptability) and PROMs (i.e., perceived functioning in real-world) were analyzed.

**Results:** The rate of adherence to HEAD treatment in clinic (≥90%) and at home (77%) was high. Usability of HEAD system was judged as good (System Usability Scale, median 70.00) in clinic and even more at home (median 80.00). Similarly, administering the Technology Acceptance Model 3 questionnaire we found high scores both in clinic/at home (Usefulness, mean 5.39 ± 1.41 SD/mean 5.33 ± 1.29 SD; Ease of use, mean 5.55 ± 1.05 SD/ mean 5.45 ± 1.17 SD, External Control, mean 4.94 ± 1.17 SD/mean 5.07 ± 1.01 SD, Relevance, mean 5.68 ± 1.29 SD/mean 5.70 ± 1.13 SD and Enjoyment, mean 5.70 ± 1.40 SD/mean 6.01 ± 1.08 SD). After ClinicHEAD, participation and autonomy in daily routine was maintained or even ameliorated (PD and stroke > MS). Whereas, increased functionality and participation in the MS group was found only after HomeHEAD intervention.

**Discussion:** Our results suggest that a tele-health-based approach is both feasible and efficient in providing rehabilitation care to CNDs from clinic to home. Increasing and maintaining participation as well as autonomy in daily routine are promising findings that open up scenarios for the continuity of care at home through DH-care for CNDs.

## Introduction

Parkinson Disease (PD), Multiple Sclerosis (MS), and Stroke are the more frequent chronic Neurological Diseases (CNDs) that can lead to significant motor and cognitive disability: worldwide data report 2.5 million people with MS (1), 7.9 to 19 individuals with PD per 100,000 person-year (2) and 5.5 million deaths due to stroke in 2016 (3). In recent years, new models of digital health (DH) enabling continuity of care are increasingly explored as new solutions to the long-term patient maintenance. Also, growing effort has been spent in the development of technology-enabled treatments, able to be carried out outside clinic setting, with promising results (4–14). Especially, telerehabilitation aids in decreasing socioeconomic costs related to these pathologies and their weight on the healthcare system (15–17). Also, technology-enabled rehabilitation at home allows people with chronic diseases to combine pathology management with their everyday social life (5).

To ensure effectiveness of tele-treatment, a continuous double loop communication between home and clinic environment is needed: in this sense, digital health care platforms constitute the central hub through which health professionals can monitor patient performance at home (18) and consequently modify treatment during the whole period of telerehabilitation. Frequency of rehabilitation and duration of treatment are important parameters that should not be overlooked. In fact, there are health care guidelines for clinical practitioners that detail the frequency and duration of rehabilitation activities specifically for different pathologies, such as MS, PD and stroke (19). For example, strength training, reported as efficacious for MS, PD and stroke patients (20–22) should be performed 2–3 days per week to reach benefits on daily living with a duration per session ranging between 10 and 40 minutes. However, little is known regarding frequency and dose treatment guidelines for treatments administered in a home-based setting.

Unfortunately, adherence to home rehabilitation protocols, including telerehabilitation, is a concern (23). People with neurological disorders that could benefit from rehabilitation often do not adhere to a prescribed protocol once they are in their home environment. This could provide serious consequences, such as loss of functioning, pain, muscle wasting etc., that are risks deriving from a lack of rehabilitation not only in an acute condition, but also in a chronic phase. Few recent studies have investigated the factors affecting adherence in order to predict and enhance adherence to telerehabilitation. An interesting work created a quantitative adherence prediction model based on baseline patients' characteristics by individualizing an important predictive role of education, satisfaction about the treatment and psychological profile (24). Another contribution investigated variation of adherence to treatment comparing different modes of cycling treatment administration, such as active or passive exercises, reporting more satisfying adherence to the passive mode of exercises (25). These contributions demonstrated a pivotal interest on the topic.

Another aspect to be considered regarding new DH approaches is the active role of the patient that is empowered and engaged in own care management, with consequences also on perceived care outcome. In particular, an “e-patients” term has been coined to highlight patients involved in decision-making and management of their own care (26). In fact, patient-reported outcome measures (PROMs) are increasingly used as real-world functioning measures that incorporate self-defined assessments of personal well-being during the management of care (27). Recent clinical practice health guidelines promote the integration of these latter measures into long-term care of patients (28).

The present study aims to report results on efficiency measures and PROMs of the Human Empowerment Aging and Disability program (HEAD), a DH-telerehabilitation system for people with chronic neurological diseases. In particular, we tested HEAD treatment during 1-month of rehabilitation program in clinic and during 3-months of HEAD telerehabilitation at home, comparing patients performances for PD, MS, and chronic stroke.

## Materials and Methods

### Participants

The study was carried out in two steps: ClinicHEAD and HomeHEAD. In the ClinicHEAD (first step) subjects with PD, MS, and chronic stroke (*N* = 107) were consecutively recruited. They were identified by the neurologists of the clinics from people that periodically receive neurological follow-up (outpatients) from the respective centers: Valduce Hospital Villa Beretta Rehabilitation Center in Lecco (*n* = 34; 17 stroke, 7 PD, 10 MS), IRCCS Don Carlo Gnocchi Foundation in Milan (*n* = 43, 12 stroke, 10 PD, 21 MS) and District Clinic San Camillo in Turin (*n* = 30, 16 stroke, 13 PD, 1 MS).

Inclusion criteria for enrollment were: [a] age range 18–80; [b] diagnosis of PD in stable treatment for at least 3 years and with a Hoehn and Yahr score ≤ 2 (29), diagnosis of MS without relapses in the last 3-months and with an Expanded Disability Status Scale [EDSS (30)] score ≤ 5.5, diagnosis of stroke in chronic phase, at least 6-months after the acute event.

Exclusion criteria for recruitment were the following: [a] Mini Mental State Examination (31) score < 20; [b] presence of disabling pain; [c] upper limb limited passive range of motion; [d] epilepsy; [e] severe deficit of visual acuity and auditory perception; [f] severe deficit in communication and severe dysmetry.

After enrollment and baseline assessment, they were consecutively assigned into the Clinic and Home HEAD programs.

All participants provided written and informed consent to take part in the study.

### The HEAD Program

The HEAD rehabilitation was conceived as a multidimensional program for the continuity of care at home for patients with chronic neurological diseases. It aimed to enhance motor and cognitive abilities such as balance, lower and upper body endurance, strength and speed, memory, executive functions, language and dual-tasking activities, in order to improve patient's everyday functional skills. Procedure and contents of each rehabilitative activity was defined by a health professionals' team including neurologists, physiotherapists and neuropsychologists. Table 1 describes all activities included in HEAD rehabilitation program.

**Table 1:** List and brief description of HEAD activities.

**Activities**	**Description**
**UPPER LIMBS WARMING-OUT**	
“Delete and Go”	*Movie stops and subject has to perform movements of arms detected by Kinect or Leap Motion Controller (LMC) in order to delete the movie screenshot that appears in the screen. When the 80% of the screenshot has been deleted the movie continues*
“Unveil and Go”	*Movie stops and subject has to perform movements of arms detected by Kinect or LMC in order to unveil the screenshot of the movie that appears covered on the screen. When the 80% of the screenshot has been unveiled the movie continues*
“Swim and Go”	*Movie stops and subject is required to perform a certain numbers of strokes detected by Kinect or LMC to continue the vision of the movie*
**LOWER LIMBS WARMING-OUT**	
“Up and Go”	*Movie stops and subject is asked to perform up-the-stairs movements detected by Kinect to climb virtual stairs in order to reach the movie screenshot that appears at the end of the stairs and let video continue*
“Goal and Go”	*Movie stops and subject has to perform a hit-the-ball movement detected by Kinect: each correct movement corresponds to a percentage of zooming of the screenshot of the movie. The maximum zoom of the screenshot let movie continue*
**UPPER LIMBS PRECISE MOVEMENT**	
“Turn pages and watch”	*Movie stops and a book with figures related to the video appears. Subject has to perform turn-pages movements detected by LMC to let movie continue*
“Grasp and Move”	*Movie stops and different images presented in the video appear on the screen. Subject has to grasp and move them in a box through hand movement detected by Kinect or LMC to let video continue*
“Pinch and Take”	*Movie stops and different images presented in the video appear on the screen. Subject has to grasp and move them in a box through pinch movement detected by LMC to let video continue*
**LOWER LIMBS PRECISE MOVEMENT**	
“Dribble and watch”	*Movie stops and subject has to juggle a ball by performing dribble-ball movements detected by Kinect sensor to let video continue*
“March and Go”	*Movie stops and subject has to perform march movements detected by Kinect in order to get nearer the screenshot of the movie, that appears far, to let video continue*
**TORSO MOVEMENT**	
“Play and watch”	*Movie stops and subject has to play drums by managing the movement of the chopstick through torso movements detected by Kinect to let video continue*
**EXECUTIVE FUNCTION**	
“Maze”	*After watching video, subject has to perform harm and torso movements detected by Kinect to guide a ball in a maze in order to reach the exit which reports the right category of the video*
“Shaky Trunk”	*After watching video, several words, both related to the movie and not related, appear on the screen. Subject has to select only the words related to the video by a grasp movement of the hand detected by Kinect. Then, different trunks on which is placed a ball appear. Subject has to manage the direction of the ball through torso movements detected by Kinect in order to let the ball fall in the box of the same color*
“Puzzle”	*Movie stops and subject has to complete a puzzle depicting a screenshot of the movie*
**MEMORY**	
“In the box”	*After watching video, subject is required to perform torso or hand movements detected by Kinect in order to manage the direction of a box while images related and not related to the movie fall up to down. Subject has to catch only images related to the movie*
“Reorder and win”	*After watching video, subject has to order a set of screenshots following the sequential order of video events through arm movements detected by LMC*
**LANGUAGE**	
“Quiz: grasp the answer”	*After watching video, subject has to solve a quiz by choosing the right answer regarding video content through hand movements detected by LMC*
**LEISURE ACTIVITIES**	
“Shaving”/ “Making-up”/“Shampooing”	*Subject has to reorder actions required for shaving/making-up/shampooing in sequence. Then, different objects appear on the screen and subject is required to grasp a certain object related with this activity. Finally, a photo of a man appears on the screen and subject has to imitate movements related to shaving/making-up /shampooing on the image by hand movements detected by LMC. After activity, movie start as a reward*
**DUAL TASK**	
“Pair symbols with similarity”	*Different images appear on the screen. Subject has to pair equal images with ratio 1:1 or 1:2 or 1:3 and move them in a box by paying attention to do not touch different images through hand movements detected by LMC. After activity, movie start as a reward*
“Pair number and object”	*Subject has to memorize an association between number and object and then pair the right number with the right object through hand movements detected by LMC. After activity, movie start as a reward*

The HEAD virtual platform represented the hub for communication between the clinic and the patient's home, allowing rehabilitation activities to be administered through a gaming setting in order to work on goal-directed movements in a virtual reality (VR) scenario. This platform was designed as a bridge between clinic and patient's home setting, making a double loop feedback possible between the two environments. Before every rehabilitation session, physiotherapists and psychologists defined the contents of the session, in the sense of type of activities, repetitions and level of difficulty through the HEAD virtual platform. In this manner, although HEAD technology allowed the same setting for each patient, contents of the rehabilitation program were tailored and personalized according to the different needs related to the pathology of the patient and the level of disability. Patients accessed the platform with their own credentials to start each telerehabilitation session and, health professionals were able to tailor rehabilitation along the whole period of treatment by remotely checking the quality of the gaming performance of the patient reported in the platform.

To run the HEAD program, a PC, internet connection and motor capture devices, such as Kinect (Microsoft, WA, USA) and Leap Motion (Leap Motion Inc., CA, USA), were needed.

The rehabilitation activity was embedded in short video clips. Each video clip lasted from 2 to 9 minutes, and was interrupted between 2 to 6 times on the basis of repetitions of the rehabilitation activity. In general, video clips had three main purposes: as motivating breaks that inter-cut the rehabilitative activities, providing emotional and cognitive stimuli regarding the rehabilitative activities, or awarding the participant at the end of the exercise. Many of the motor and cognitive exercises were directly related to the video. Thus, participants had to erase an image just seen, by means of large movements of the arms in order to continue watching the clip. Alternatively, participants were asked to order the sequences of the film clip just seen, or had to answer questions about the content of the film clip. Patients thus actively controlled their viewing of the movie clips and their progression.

Each activity ended with a feedback of the results, according to an algorithm based on the percentage of completion, number of errors and duration of the performance. The scoring was illustrated by stars, with a minimum of 1 to a maximum of 5 stars being awarded.

During the month of treatment in clinic, the HEAD program was supervised by physiotherapists and neuropsychologists and was administered through 45 min sessions 3 times per week. Once at home, the HEAD program was carried out five consecutive days per week, 30–45 min each session.

Each participant accessed the HEAD portal through his personal credentials to perform his own individual daily program consisting of 3–6 neuromotor activities according to his needs. The participants were free to choose the time of day and weekdays in which to carry out the activities. Finally, participants had the opportunity of calling the Help Desk Service or therapists for technical problems or related issues. A phone call by therapists to participants was planned once a week to check for patient compliance.

### Measures

All patients recruited were administered a rehabilitation with VR technology for 1-month in clinic (Time 1: ClinicHEAD). After rehabilitation in clinic, patients were consecutively allocated to HEAD telerehabilitation at home for 3-months (Time 2: HomeHEAD) with a ratio of 1:3. This ratio was due to the limited availability of the HEAD technological kits. For this reason, one patient each three was allocated to continue treatment for 3-months at home. In the second step of the study the participants not allocated to the HomeHEAD group were asked to not participate in physical activities different from those that they would usually do during the protocol duration (control group). Subsequent contributions will report efficacy of HEAD treatment based on outcome measures in each CND included in the study.

Efficiency and Patient-Reported Outcome measures were collected after ClinicHEAD rehabilitation (Time 1) and after 3-months of HomeHEAD treatment by clinicians blind to the treatment allocation (Time 2).

Part of data were obtained through questionnaires administered by a psychologist, while remaining data were extracted from the HEAD platform.

The present work, registered as Clinical Trial ID: NCT03025126, provides preliminary data on efficiency of the HEAD protocol.

#### Baseline Assessment

Patients recruited were screened with:

1. Montreal Cognitive Assessment [MoCA (32)] as a measure of global cognitive functioning. Conti's correction was adopted to transform scores on the basis of age and years of education of people. This tool allowed a brief screening of cognitive level evaluating different domains: attention, executive functions, memory, language, visual-spatial abilities, abstraction, calculation, and orientation. Score range is 0–30, with a maximum score of 30.
2. 2 Minute Walk Test [2MWT (33)] for a quantitative analysis of gait endurance. Participants were instructed to walk as far as possible over 2 minutes and the distance covered was collected.
3. 10 Meter Walk Test [10MWT (34)] to measure gait speed. Participants were required to walk 10 meters while time was measured. The score was obtained by dividing the distance by the time spent to cover it.

#### Output Measures

System Usability Scale [Brooke (35)] was administered for a measure of perceived easiness of use of the HEAD system. This is a 10-item, 5 point Likert scale (1 = strongly disagree, 5 = strongly agree). Scoring instructions of Brooke (35) were considered. The final score ranges from 10 to 100. A cut-off score, indicating a satisfying level technological system's usability, is 68. Learnability and usability sub-scores were also obtained in accordance to Lewis and Sauro's indications (36, 37).

Adherence to treatment in clinic and at home was calculated by extracting the following indexes from output of the platform: percentage of total sessions performed, mean number of activities performed per session, mean duration of activities performed per session. We analyzed the same indexes singularly per each week of telerehabilitation at home. Additionally, we analyzed the number of sessions per week performed considering 3 session/week as the recommended frequency of rehabilitation, following Kim's et al. (19) indications. We considered as drop-out participants who followed <50% of treatment period in clinic (<2 weeks of treatment) and at home (<6 weeks of treatment).

Technology Acceptance Model-3 (38) was utilized in order to deepen patients' beliefs related to their inclination to experience the HEAD system. This scale, in fact, specifically explores the perceived ease of use, such as the degree of difficulty that the use of a technology system involves, and the perceived usefulness, as the belief that the use of a specific technology system allows improving one's own productivity. For the purpose of the present study, we focus our analysis only on determined domains of the scale: Perceived usefulness, perceived ease of use, perceptions of external control, perceived enjoyment, Job relevance. Response on a 7-point Likert scale were collected (Totally Disagree = 1/ Totally Agree = 7).

Finally, an *ad-hoc* questionnaire was created with the purpose to investigate specific barriers patients experienced during rehabilitation at home. This tool was additionally administered to patients recruited in Don Gnocchi Foundation for a deepened investigation. The questionnaire was composed by 11 items with a 5-point Likert scale (0 = Absolutely not/Never, 4 = Very much/Always) (see Table S1 to consult the tool). The questionnaire was scored grouping items into 5 groups in order to obtain 5 total indexes related to crucial aspects to investigate barriers encountered during experience of telerehabilitation. In particular, we analyzed answers extracting the following indexes: (1) motivation (mean score of item 6 and 8) [*Did you need support of other persons (ex. your son, bride/wife…) to be motivated to perform HEAD activities (did they remind you to do them?)/ When I didn't perform activities it was because I wouldn't*], (2) logistics (mean score of item 1 and 2) [*Did having HEAD system at home bother you?”*/“*How much did you need to modify arrangement of furniture to place HEAD technology devices?*”], (3) autonomy (mean score of item 5 and 6) [*Did you need support of other persons (ex. Your son, bride/wife…) to prepare HEAD technology setting?*/ *Did you need support of other persons (ex. your son, bride/wife…) to perform HEAD activities?*], (4) inclusion in the routine (mean score of item 3, 4 and 9) [*Did you modify your routine to include HEAD activities during the week (ex. Did you eat earlier than usual? Did you stop to have nap after lunch?)*/*Did you renounce to perform other activities to do HEAD program?*/ *When I didn't perform activities it was because I couldn't*], (5) technical problems (mean score of item 10 and 11) [*When I didn't perform activities it was because the system did not work/When I didn't perform activities it was because even if the system worked, the internet connection did not*].

#### Patient-Reported Outcome Measures

Two categories of International Classification of functioning [ICF (39)] on activities and participation in daily life were considered as PROMs: Carrying out daily routine (d230) and Recreation and leisure (d920). These categories were extracted by administering the item 3 of EuroQoL EQ-5D-5L (40–43) and the item 12 of Short Form 12 health survey questionnaire [SF12 (44–47)], respectively. These two items were then translated in the 5 ICF qualifiers. Specifically, we associated ICF qualifier 4 (complete problem) to answer “All the time/Extremely,” qualifier 3 (severe-complete problem) to answer “Most of the time/a good bit of the time/Quite bit,” qualifier 2 (moderate-severe problem) to answer “Some of the time/Moderately,” qualifier 1 (mild-moderate problem) to answer “A little of the time/a little bit” and qualifier 0 (no problem) to answer “Never/Not,” following previous mapping works (44, 46).

### Statistical Analysis

Statistical analysis was performed using MedCalc^®^ Software Version 15.2.1.

Normal distribution of variables was checked through Kolmogorov-Smirnov normality test. According to this test, parametric or non-parametric analysis were performed for the comparison among three pathology groups (PD vs. MS vs. stroke).

To analyze efficiency measures such as adherence to HEAD treatment, usability and acceptance of the HEAD system, descriptive statistics were run in each pathology group. Comparison among groups was also reported through ANCOVA or General Linear Model, by covarying for recruitment center. Bonferroni *post-hoc* test was considered. To analyze *ad-hoc* questionnaire results we performed descriptive statistics.

To analyze PROMs, we reported the distribution of qualifiers (percentages) of the ICF categories at the different time points (Enrollment T0, post ClinicHEAD T1, and post HomeHEAD T2). Then, patients were classified into delta score ≤ 0, indicating a perception of maintenance or amelioration over time, and delta score > 0, referring a perception of worsening over time. Percentages of sample reporting delta score ≤ 0, reported as stable/ameliorated patients, and delta score > 0, as worsened patients, were calculated and Chi-square χ^2^ was performed. Results were considered statistically significant when *p* < 0.05.

## Results

### Participants

Table 2 shows demographic characteristics of sample included in the study.

**Table 2:** Summary statistics and comparison results of patient groups at baseline.

	**PD**	**MS**	**Stroke**	**Comparison [Test (*p*)]**	**All**
***TIME 1: ClinicHEAD***					
*N*	30	32	45	–	107
Age (M ± sd)	66.30 ± 8.77	52.75 ± 10.62	61.04 ± 13.25	11.29 (<0.001)	60.04 ± 12.43
Sex (M:F)	16:14	15:17	26:19	0.89 (0.640)	57:50
Education	11.73 ± 4.40	11.22 ± 3.25	12.44 ± 4.12	0.92 (0.401)	11.88 ± 3.96
MoCA	23.60 ± 3.41	23.69 ± 3.19	21.71 ± 4.79	3.05 (0.051)	22.83 ± 4.08
2MWT	131.66 ± 37.30	92.36 ± 34.11	81.94 ± 43.91	13.72 (<0.001)	100.77 ± 44.44
10MWT	1.41 ± 0.52	0.98 ± 0.52	0.84 ± 0.47	10.55 (<0.001)	1.07 ± 0.55
***TIME 2: HomeHEAD***					
*N*	11	14	13	–	38
Age (M ± sd)	65.55 ± 9.06	51.93 ± 8.76	57.77 ± 17.17	3.73 (0.034)	57.87 ± 13.25
Sex(M:F)	4:7	7:7	6:7	0.48 (0.112)	17:21
Education	11.27 ± 4.69	12.07 ± 3.25	14.85 ± 4.00	2.79 (0.075)	12.79 ± 4.15
MoCA	22.91 ± 3.21	23.85 ± 3.74	22.62 ± 5.44	0.20 (0.819)	23.13 ± 4.20
2MWT	131.64 ± 38.94	93.96 ± 31.57	79.54 ± 26.22	7.31 (0.003)	101.27 ± 38.36
10MWT	1.43 ± 0.59	1.04 ± 0.47	0.83 ± 0.34	4.48 (0.019)	1.09 ± 0.52

*2MWT, 2 minutes walk test; 10MWT, 10 meters walk test; M, mean; MoCA, Montreal Cognitive Assessment; MS, multiple sclerosis; N, number; PD, Parkinson Disease; sd, standard deviation*.

*ClinicHEAD:* All patients enrolled in the study (*n* = 107) followed a program of 12 sessions of HEAD rehabilitation in clinic. A total of 107 patients with CNDs was composed of 30 people with PD, 32 with MS and 45 with chronic stroke. The three groups were comparable in terms of gender distribution (χ^2^ = 0.89, *p* = 0.640) and years of education (F = 0.92, *p* = 0.401) while there was a statistically significant difference in age between MS and the other two groups (F = 11.29, *p* < 0.001). In terms of global cognitive level, we found a trend for a lower MoCA score in stroke patients compared to those with MS or PD (F = 3.05, *p* = 0.051). Finally, endurance and velocity assessed through 2MWT and 10MWT scores were significantly higher in PD than in other patients' groups (2MWT: F = 13.72, *p* < 0.001; 10MWT: F = 10.55, *p* < 0.001).

*HomeHEAD:* Thirty-eight patients were then allocated to HomeHEAD treatment after ClinicHEAD period to test the system for the continuity of care at home. In particular, 11 PD, 14 MS, 13 stroke were assigned to telerehabilitation at home. Similarly to the ClinicHEAD sample, patients groups were comparable for gender distribution (χ^2^ = 0.479, *p* = 0.112) and education (F = 2.79, *p* = 0.075), but not for age: we reported a statistically significant difference between PD and MS group (F = 3.73, *p* = 0.034). The three groups did not differ in MoCA score. Instead, endurance assessed through 2MWT was significant higher in PD than in other patients' groups (F = 7.31, *p* = 0.003) and velocity assessed through 10MWT was major in PD than stroke (F = 4.48, *p* = 0.019).

The group enrolled for *ClinicHEAD* and the sub-group allocated to *HomeHEAD* did not significantly differ in gender distribution (χ^2^ = 1.42, *p* = 0.116), age (F = 2.00, *p* = 0.160), education (F = 2.33, *p* = 0.130), global cognitive level (F = 0.15, *p* = 0.698), endurance (F = 0.03, *p* = 0.868), and velocity (F = 0.33, *p* = 0.569).

### Efficiency Measures Results

In Table 3 we report data on efficiency measures after ClinicHEAD (Time 1) and after HomeHEAD (Time 2), in the three pathologies.

**Table 3:** Efficiency measures results of treatment in clinic.

	**PD**	**MS**	**Stroke**	**Comparison [Test(*p*)]**	**All**
***TIME 1: ClinicHEAD***					
*N*	30	32	45	–	107
Adherence	0.93 ± 0.11	0.90 ± 0.18	0.94 ± 0.10	1.23(0.296)^*^	0.92 ± 0.13
**Duration of treatment (M** **±** **sd)**					
Number of activities	4.54 ± 1.70	4.97 ± 1.39	4.16 ± 0.88	0.90(0.408)^*^	4.51 ± 1.34
Duration of session, min	39.62 ± 18.14	43.70 ± 18.54	36.81 ± 13.05	0.40(0.675)^*^	39.65 ± 16.42
SUS (Median, 25–75 percentile)	68.75, 60.00–82.50	75.00, 62.50–84.38	70.00, 62.50–80.63	0.77 (0.679)^§^	70.00, 62.50–82.50
Usability	2.88, 2.63–3.29	3.14, 2.75–3.43	3.00, 2.63–3.38	1.64 (0.440)^§^	3.00, 2.63–3.43
Learnability	3.00, 1.50–3.50	2.50, 1.50–3.50	2.50, 1.50–3.50	0.99 (0.605)^§^	2.50, 1.50–3.50
**TAM3 (M** **±** **sd)**					
Usefulness	5.54 ± 1.36	5.08 ± 1.58	5.50 ± 1.31	2.82(0.065)^*^	5.39 ± 1.41
Ease of use	5.59 ± 0.92	5.53 ± 1.09	5.55 ± 1.12	0.28(0.760)^*^	5.55 ± 1.05
External control	5.05 ± 0.92	4.93 ± 1.39	4.86 ± 1.15	0.77(0.466)^*^	4.94 ± 1.17
Relevance	5.84 ± 1.51	5.30 ± 1.40	5.84 ± 0.99	1.37(0.260)^*^	5.68 ± 1.29
Enjoyment	5.67 ± 1.33	5.91 ± 1.02	5.56 ± 1.66	0.47(0.629)^*^	5.70 ± 1.40
***TIME 2: HomeHEAD***					
*N*	11	14	13	–	38
Adherence	0.86 ± 0.14	0.66 ± 0.28	0.82 ± 0.15	6.00(0.007)^*^	0.77 ± 0.22
**Duration of treatment (M** **±** **sd)**					
Number of activities	3.88 ± 0.90	4.03 ± 0.83	3.24 ± 0.42	0.81 (0.457)^*^	3.72 ± 0.80
Duration of session, min	37.74 ± 7.61	37.57 ± 9.89	30.66 ± 5.67	0.72 (0.497)^*^	35.17 ± 8.47
SUS (Median, 25–75 percentile)	85.00, 77.50–92.50	67.50, 55.00–85.00	80.00, 68.13–84.38	3.14 (0.205)^*^	80.00, 67.50–85.00
Usability	3.36, 3.25–3.71	2.86, 2.42–3.43	3.13, 2.65–3.50	2.46 (0.292)^*^	3.20, 2.57–3.50
Learnability	4.00, 2.50–4.00	2.50, 1.50–3.63	3.00, 2.63–3.88	3.06 (0.195)^*^	3.00, 2.00–4.00
**TAM3 (M** **±** **sd)**					
Usefulness	5.25 ± 1.97	5.23 ± 1.11	5.53 ± 0.10	1.47(0.254)^*^	5.33 ± 1.29
Ease of use	4.68 ± 1.10	5.75 ± 1.21	5.65 ± 1.01	2.18(0.139)^*^	5.45 ± 1.17
External control	4.89 ± 0.45	4.95 ± 1.27	5.38 ± 0.95	0.50(0.613)^*^	5.07 ± 1.01
Relevance	5.62 ± 1.50	5.50 ± 1.10	6.04 ± 0.90	0.50(0.616)^*^	5.70 ± 1.13
Enjoyment	6.00 ± 1.29	5.97 ± 0.10	6.06 ± 1.14	0.97 (0.396)^*^	6.01 ± 1.08

*M, mean; MS, multiple sclerosis; N, number; PD, Parkinson Disease; sd, standard deviation; SUS, System Usability Scale; TAM3, Technology Acceptance Model-3*.

* ANCOVA comparison was performed co-varying recruiting center, age, gender, education;

§ *Kruskall-Wallis test was performed*.

After ClinicHEAD we found a high level of adherence to treatment in all three patients' groups (mean score of all sample: 0.92 ± 0.13) with no significant differences among patients' groups (F = 1.23, *p* = 0.296). In terms of duration of treatment, as number of activities per session and minutes per session, each session consisted of about 40 minutes multidimensional treatment and was composed of about 4–5 activities. We did not find differences among groups in number of activities (F = 0.40, *p* = 0.675) and minutes of session (F = 0.90, *p* = 0.408). We registered only 1 drop out. In terms of perceived usability of the HEAD system in clinic, we reported a good level of usability, with a median SUS score of 70.00 in all patients' groups with no differences among pathologies (F = 0.77, *p* = 0.679). Finally, considering sub-domains of TAM3, such as perceived system Usefulness, Ease of use, External control, Relevance and Enjoyment, data of all groups supported the high level of functionality of HEAD technology (Usefulness: mean score 5.39 ± 1.41; Ease of Use: mean score 5.55 ± 1.05; External Control: mean score 4.94 ± 1.17), the perceived treatment efficacy (Relevance: mean score 5.68 ± 1.29) and the motivating aspects of HEAD contents (Enjoyment: mean score 5.70 ± 1.40). No differences among patients' groups were registered in all TAM3 subscores (Usefulness: F = 2.82, *p* = 0.065; Ease of Use: F = 0.28, *p* = 0.760; External control: F = 0.77, *p* = 0.466; Relevance: F = 1.37, *p* = 0.260; Enjoyment: F = 0.47, *p* = 0.629).

At HomeHEAD (Time 2), we registered 7.89% of drops in the whole group. In general, we reported a discrete adherence to treatment at home (mean score in whole group: 0.77 ± 0.22). More specifically, we observed a better adherence to treatment in PD and stroke groups than in the MS group (F = 6.00, *p* = 0.007). Focusing on duration of treatment, we did not find group differences in the number of activities performed per session (F = 0.81, *p* = 0.457) nor in the length of treatment per session (F = 0.72, *p* = 0.497).

Patient assessment of system usability, as shown by SUS score, was higher in the PD group (median: 85.00) compared to stroke (median: 80.00) and MS (median: 67.50) groups. In general, usability of the system at home was estimated as good (median of whole group: 80.00). No differences among pathologies groups were reported (F = 3.14, *p* = 0.205).

Results of TAM3 questionnaire highlighted high level of functionality of the system at home in all domains explored (Usefulness: 5.33 ± 1.29; Ease of Use: 5.45 ± 1.17; External Control: 5.07 ± 1.01; Relevance: 5.70 ± 1.13; Enjoyment: 6.01 ± 1.08), suggesting that patients perceived HEAD program as useful, easy to use, intuitive, relevant for everyday life and playful. Again, patients' groups did not differ in TAM3 subscores (Usefulness: F = 1.47, *p* = 0.254; Ease of Use: F = 2.18, *p* = 0.139; External Control: F = 0.50, *p* = 0.613; Relevance: F = 0.50, *p* = 0.616; Enjoyment: F = 0.97, *p* = 0.396).

Additionally, adherence to single sessions at home for all period of telerehabilitation (60 sessions, 5 sessions/week) was observed. In particular, we analyzed adherence to sessions in each single week of treatment in each pathology group, considering percentage of adherence to session in each week (1 = adherence to 5 sessions/week; 0 = adherence to 0 sessions/week).

Table 4 reports percentage of adherence to sessions in each week (1 = adherence to 5 sessions/week; 0 = adherence to 0 sessions/week) and the mean number of activities per session in each of 12 weeks of treatment at home.

**Table 4:** Total adherence to HEAD telerehabilitation along 3-months of treatment at home.

	**W1**	**W2**	**W3**	**W4**	**W5**	**W6**	**W7**	**W8**	**W9**	**W10**	**W11**	**W12**
**Adherence %**												
PD	0.64	**0.80**	**0.87**	**0.96**	**0.93**	**0.93**	**0.93**	**0.89**	**0.84**	0.78	**0.80**	0.67
MS	0.66	0.79	**0.88**	**0.83**	0.68	0.67	0.71	0.69	0.65	0.57	0.55	0.43
Stroke	**0.80**	**0.92**	**0.92**	**0.92**	**0.90**	**0.87**	**0.88**	0.79	0.74	0.77	0.62	0.57
Whole group	0.71	**0.84**	**0.89**	**0.89**	**0.82**	**0.81**	**0.83**	0.78	0.74	0.69	0.64	0.55
**Activities/session**												
PD	3.73	4.14	3.64	4.09	3.55	3.95	4.02	3.98	4.03	4.51	4.47	4.23
MS	4.12	4.26	4.04	4.15	4.05	4.20	4.24	4.27	4.18	4.35	4.27	4.58
Stroke	3.52	3.58	3.60	3.35	3.26	3.35	3.25	3.20	3.13	3.00	2.65	2.94
Whole group	3.78	3.97	3.77	3.84	3.59	3.79	3.80	3.80	3.74	3.87	3.77	3.85

*MS, multiple sclerosis; PD, Parkinson Disease; W, week; %, percentage. Adherence > 80% is reported in bold*.

Based on recent data on recommended frequency of treatment to guarantee rehabilitation effectiveness (19), we considered 0.60 as the ideal adherence per week (3 sessions/5 per week = 1.00). We reported a good adherence (more than 88%) of PD group from the second to the eleventh week of treatment. Stroke group showed an adherence > 80% from the first to the eighth week of treatment, followed by a discrete adherence (75%) in the ninth and tenth week. MS group, instead, demonstrated a discontinuous adherence to treatment, by reported an adherence >85% only from second to forth week. Overall, the whole group presented a good adherence (>82%) from the second to the eighth week of treatment (for details, see Table S2).

*Ad-hoc* questionnaire on barriers possibly experienced at home reported positive data.

People reported to have encountered very few barriers during their experience of HEAD telerehabilitaiton. In particular, all participants were motivated to carry out rehabilitation as mean score of motivation barriers was 0.03 ± 0.13. Also, from a logistical point of view, the presence of the technological kit in one's home was not perceived as burdensome, as suggested by the low mean score of logistical barriers: 0.31 ± 0.36. Another positive result was that people could perform activities in autonomy: although sometimes preparation of technological setting needed help from the caregiver, performing activities did not. In fact, we collected a mean score of autonomy barriers of 0.31 ± 0.44. Furthermore, HEAD rehabilitation resulted well-integrated into patients' routine: we reported a mean score of 0.40 ± 0.30 at inclusion in the routine barriers. Finally, technological problems represented the only barrier that sometimes impeded the performance of activities: mean score of technological issues was 0.94 ± 0.73.

### Patient-Reported Outcome Measures Results

The whole sample was classified as moderate-to-severe in autonomy in daily routine (d230 ICF domain) and as mild-to-moderate in socialization (d920 ICF domain) at baseline.

We report in Table 5 percentages of patients who perceived HEAD treatment as successful in their daily living (daily routine and socialization) and who did not, by reporting a change of level of autonomy and socialization in daily life between baseline and after HEAD treatment in all group that experienced ClinicHEAD (Time 1) (*n* = 107) and in the sub-group who additionally experienced HomeHEAD (Time 2) (*n* = 38). We considered as stable or ameliorated patients who presented an ICF score change between time points = 0 or ≤ 1, such as a perceived successful effect of HEAD treatment. On the contrary, patients were considered as worsened when they reported an ICF score change ≥ 1.

**Table 5:** Changes in autonomy (d230) and participation (d920) after ClinicHEAD and after HomeHEAD.

	**ICF category**	**T**	**ICF qualifier (%)**					**T1 vs. T0**			T2 vs. T0^*^		
			**No problem**	**Mild problem**	**Moderate problem**	**Severe problem**	**Complete problem**	**Stabilization/amelioration %**	**Worsening %**	***p***	**Stabilization/amelioration %**	**Worsening %**	***p***
PD	d230	0	23.3	20.0	40.0	16.7	0.0	0.97	0.03	**<0.001**	1.00	0.00	–
		1	33.3	30.0	26.7	10.0	0.0						
		2^*^	36.4	45.5	9.1	9.0	0.0						
	d920	0	33.3	16.7	33.3	16.7	0.0	0.83	0.17	**<0.001**	0.80	0.20	0.114
		1	37.9	20.7	27.6	13.8	0.0						
		2^*^	36.4	18.2	27.3	18.1	0.0						
MS	d230	0	22.6	19.4	35.5	19.4	3.1	0.59	0.41	0.441	0.89	0.11	**0.045**
		1	13.9	17.2	44.8	17.2	6.9						
		2^*^	25.0	41.7	33.3	0.0	0.0						
	d920	0	30.0	26.7	20.0	20.0	3.3	0.63	0.37	0.359	0.82	0.18	0.070
		1	24.2	17.2	37.9	20.7	0.0						
		2^*^	33.3	33.3	16.7	16.7	0.0						
STROKE	d230	0	11.1	37.8	26.7	17.8	6.6	0.74	0.26	**0.002**	0.82	0.18	0.070
		1	18.3	29.5	29.5	18.2	4.5						
		2^*^	16.7	33.3	33.3	16.7	0.0						
	d920	0	31.8	20.5	22.7	22.7	2.3	0.82	0.18	**<0.001**	0.73	0.27	0.228
		1	35.6	15.6	26.7	15.6	6.5						
		2^*^	33.4	8.3	33.3	25.0	0.0						
ALL	d230	0	17.9	27.4	33.0	17.9	3.8	0.77	0.23	**<0.001**	0.90	0.10	**<0.001**
		1	21.4	26.2	33.0	15.5	3.9						
		2^*^	25.7	40.0	25.7	8.6	0.0						
	d920	0	31.7	21.2	25.0	20.2	1.9	0.79	0.21	**<0.001**	0.78	0.22	**0.003**
		1	33.0	17.5	30.1	16.5	2.9						
		2^*^	34.3	20.0	25.7	20.0	0.0						

*d230, Carrying out daily routine; d920, Recreation and participation; ICF, International Classification of Functioning; MS, Multiple Sclerosis; PD, Parkinson Disease*.

* *, HomeHEAD group (n = 38); %, percentage. p_s_ < 0.05 are reported in bold*.

We did not find differences for T1-baseline measures between ClinicHEAD and HomeHEAD group (d230: *p* = 0.476; d920: *p* = 0.278).

*ClinicHEAD:* Results showed a high percentage of people with PD (97%) who reported a perceived maintenance or amelioration of functioning in daily life after 12 sessions of HEAD treatment in clinic. Also, a high percentage of patients with stroke judged a positive influence of HEAD treatment on participation in daily living (82%) and a discrete part of the group extended this perception to performance in daily routine (74%). We didn't find similar results in MS group, in which only 59% of patients perceived a successful effect of treatment. In general, a significantly higher number of people who perceived treatment as successful on daily life functioning was registered than people who did not (*p* < 0.001). This latter result appeared evident in PD and stroke groups, but not in the MS group.

*HomeHEAD:* We observed satisfying results in the PD group after experience at home. In fact, the entire group (100%) judged a successful effect of HEAD on daily routine and 80% of the sample referred to same perception in daily participation after telerehabilitation. Also the stroke group, which showed positive reported outcome after ClinicHEAD rehabilitation, indicated positive results after rehabilitation at home, with 82% of patients registering a positive effect of telerehabilitation on daily routine and 73% reporting benefits also on participation. Interestingly, the MS group, reported positive results in more than 80% of the group in both daily routine and participation after telerehabilitation at home. In general, we found a significant number of patients reporting positive effect of HEAD treatment on daily functioning (d230: *p* < 0.001; d920: *p* = 0.003).

## Discussion

We tested efficiency of HEAD, a new technology enabled rehabilitation program for the continuity of care at home for people with CNDs, such as PD, MS, and chronic stroke based on key performance indicators. In particular, we explored HEAD usability and acceptability together with patient's adherence to treatment, and PROMs, as perceived functioning in routine and participation in daily life. We observed output and outcome measures in clinic for a training duration of 1-month (12 sessions) and in continuity of care at home for a total duration of 3-months (60 sessions).

Our results highlighted a very high compliance rate in all three patient groups in clinic, with 92% adherence to treatment. This data is extremely relevant given that Evidence-Based Medicine guidelines reported 80% as minimum rate of adherence needed to appraise quality of clinical trials (42). In particular, we found that stroke patients followed on average 94% of sessions, whereas the numbers were 93 and 90% for PD and MS, respectively. This positive data can be explained by two main factors. First, the ease of use of the technological system can have affected participation to treatment. In fact, from the point of view of usability, our patients judged the system in clinic as efficient. This is particularly important, since the potential effect of DH is strictly related to the perceived ease of use of health care systems (43, 46). Also, investigating perceived acceptability together with usability of the system we reported satisfying feedbacks regarding HEAD technology acceptance in all three pathologies. Especially, facility of use, usefulness of the program, a good control of the system, perception of relevance on their everyday life and enjoyment was observed. Second, the modality of implementation of activities can have influenced motivation to treatment. Especially, HEAD activities were presented in a VR setting and recent evidence has supported the role of VR in influencing outcome by adhering to basic principles of rehabilitation such as intensity, environment, bio-feedback and motivation (47). Moreover, each activity was embedded in a multimedia content, consisting of short video clips, thought to be motivating for patients. Video clips have historically been used to elicit emotion and motivate people, and their dynamic nature appears to be useful in eliciting interest as well as providing an optimal artificial model of reality (48–50).

Having demonstrated the efficiency of the HEAD program in clinic, we focused also on efficiency of the system during telerehabilitation at home for 3-months. Usability and acceptance of HEAD system at home was high in the whole sample, suggesting a good functionality of the system in telerehabilitation. The high score of sub-domain “Enjoyment” of TAM-3 supported the motivating feature of the rehabilitation activities, probably due to variability of the contents included in activities during 3-months of rehabilitation. Moreover, the game-setting of the rehabilitative activities probably played a crucial role in enjoyment and consequently in acceptance of DH-treatment. Accordingly, a recent study demonstrated that game elements affect duration of enjoyment during motor exercises (51). Also, focusing on adherence, <8% of patients did not complete the entire treatment program. This is an important result since continuity of care is crucial in order to do not lose functional recovery after discharge to home (52, 53). However, while the mean adherence to treatment was over 80% in all 3 patients' groups in the first 2-months, it was lower in the last month of the program. This may be due to the high intensity of the home program: the HEAD program was administered 5 sessions/week for 3-months while Kim's et al. (19) work indicated a maximum of 2–3 sessions per week in the MS population. When considering 3 sessions/week as ideal adherence over time, we found a globally longer persistence over telerehabilitation weeks, with, for example, high adherence over 11 weeks/12 in PD group. Moreover, we observed different patterns of adherence in distinct pathologies: with a better adherence in PD and stroke than in MS. This lower adherence to treatment of the MS group might be due to the fatigue that this population experiences during treatment and in daily life (54). It is known that MS patients are faced with elevated challenges when following long-term intervention and there is an urgent need of future research focusing on solutions for continuity of care and exercise persistence in the MS population (55, 56). Moreover, we considered the implication of the video contents included in HEAD activities: movie clips were collected from historically famous movies of years 1940–1990 (obtained by RAI, Italian Radio Television s.p.a.) with the purpose to stimulate positive memories of patients and as such they may have been more targeted to an older audience. The younger age of the MS group compared to the other two pathologies may have contributed to the perception of these contents as not engaging enough. This result stresses the importance of tailoring contents of rehabilitation to age of population targeted, further studies are needed to better elucidate this issue.

A DH approach is not without barriers. Moving the rehabilitation context outside the clinic can result in difficulties for patients to manage technology systems on their own and include treatment in their daily routine. We evaluated the frequency of patients' experience of the most common barriers during 3-months of tele-treatment. Patient feedback suggested that all participants were highly motivated, that they included HEAD program in their routine, did not encounter logistical problems and were able to conduct rehabilitation activities in autonomy. The overall positive judgments support the suitability of DH in a context of global transition of CNDs rehabilitation treatment from inside to outside the clinic (57). The only barriers encountered were technology problems, probably due to the innovative features of the technology system. This is likely an obstacle that will be solved in the near future since technology transformation over time will lead to increasingly more tailored and useable systems.

Moving from efficiency to focus on PROMs enabled us to deepen our understanding of patient perceptions regarding effectiveness of treatment on their functioning in everyday life. Recent works recommended the application of these measures as extremely informative (27), especially in the investigation of effects of newly implemented DH treatments that often are conducted in a home-based context. In the present study a consistent number of subjects referenced a positive effect of treatment particularly on performance of activities in daily life (90%) and socialization (78%). Interestingly, we found a maintenance or amelioration of functioning in daily living especially high after treatment at home, with a greater number of patients reporting positive effect on autonomy in their routine after telerehabilitation at home vs. rehabilitation treatment in clinic (90% of subjects vs. 78%). This evidence is crucial, in accordance with suggestion of Steinhubl et al. (58), who, reporting definitions and scenarios fostered by the new mobile health technologies, declare that changes in the care environment are able to provide better outcomes. More importantly, this result is in line with the main goal of rehabilitation itself, that aims at the recovery of the patient's functioning in terms of its utilization in daily living; all in the overall context of the biopsychosocial digital model that foresees a collaboration between clinicians and patient facilitated by DH in the social context of the person (9). Considering socialization in daily life, we found different outcome in the three pathologies. There was a better perception of socialization after the in-clinic HEAD program than following the home telerehabilitation program. On the contrary, MS patients showed an increase in socialization after telerehabilitation at home compared to the clinic. This result may be related to the phenomenon of the baby boomer generation: people born between 1946 and 1964 need to be introduced to technology. Instead, younger people, nearer to millennials, are more familiar with technology systems. Hence, our results could be explained by demographic factors of our population targets: patients with an older age, such as those with PD and stroke, appreciated HEAD potential benefit on socialization more in clinic setting than at home, while younger people, such as the MS sample in our study, are able to report treatment potentiality on their daily life when it is fostered in their home setting.

Targeting three pathologies, MS, PD, and Stroke in this study leads to smaller sample size for each group which could constitute a limitation. However, one of the hallmarks of the proposed study is its adaptability to different functional levels and its suitability for different neurological disorders. In fact, one of the problems other studies have faced in the past is the narrow applicability of their intervention systems (59). The HEAD protocol is developed to overcome this problem. This study proposes to investigate the responsiveness of the tested intervention to the needs of diverse populations with chronic neurological pathologies that are typically seen in rehabilitative practices and need to have access to monitored continuity of rehabilitative activities.

While the sample size of this study is too small to draw conclusive evidence of the efficacy of the proposed intervention, the results support HEAD as a useful and acceptable DH-care system for people with CNDs with positive impacts on the perceived benefits for autonomy and daily life involvement. This is important given the crucial role technology will play in future neurorehabilitation models. Our findings support the notion that intensity and duration of long-term intervention must be tailored to the individual, taking into account also the personalization of contents to maintain an adequate level of engagement in rehabilitation at home.

## Data Availability Statement

The datasets generated for this study are available on request to the corresponding author.

## Ethics Statement

The studies involving human participants were reviewed and approved by the Ethics Committees of IRCCS Don Gnocchi Foundation, of inter-company of the province of Lecco, Como and Sondrio, and of inter-company “Città della Salute e della scienza” of Turin. The patients/participants provided their written informed consent to participate in this study.

## Author Contributions

FB, FM, and MS conceived the study. CG, JJ, and PG carried out the study. CC, CP, and GP collected data. CP, SI, and FB performed statistical analysis and interpreted results. FB, JJ, and SI wrote the manuscript. All authors reviewed and approved the final manuscript.

### Conflict of Interest

The authors declare that the research was conducted in the absence of any commercial or financial relationships that could be construed as a potential conflict of interest.
