# Supplementary material for: Efficiency and Patient-Reported Outcome Measures From Clinic to Home: The Human Empowerment Aging and Disability Program for Digital-Health Rehabilitation
Source: Front Neurol. 2019 Nov 19;10:1206. doi: 10.3389/fneur.2019.01206 (PMC6882300; doi:10.3389/fneur.2019.01206)
Supplement: Supplementary file 1 [file Table_1.docx]

**SUPPLEMENTARY MATERIALS OF THE PAPER:**

**Efficiency and Patient-Reported Outcome Measures from clinic to home: the Human Empowerment Aging and Disability program for digital-health rehabilitation**

1. **Table S1.** *Ad-hoc* questionnaire on barriers experienced during rehabilitation at home.

|  | Item | *Answer* | | | | |
| --- | --- | --- | --- | --- | --- | --- |
| 1 | Did having HEAD system at home bother you? | *Absolutely not* | *A few* | *Neither yes nor not* | *Moderately* | *Very much* |
| 2 | How much did you need to modify arrangement of furniture to place HEAD technology devices? | *Absolutely not* | *A few* | *Neither yes nor not* | *Moderately* | *Very much* |
| 3 | Did you modify your routine to include HEAD activities during the week (e.g. Did you eat earlier than usual? Did you stop to have nap after lunch?) | *Absolutely not* | *A few* | *Neither yes nor not* | *Moderately* | *Very much* |
| 4 | Did you renounce to perform other activities to do HEAD program? | *Absolutely not* | *A few* | *Neither yes nor not* | *Moderately* | *Very much* |
| 5 | Did you need support of other persons (e.g. Your son, bride/wife…) to prepare HEAD technology setting? | *Absolutely not* | *A few* | *Neither yes nor not* | *Moderately* | *Very much* |
| 6 | Did you need support of other persons (e.g. your son, bride/wife…) to be motivated to perform HEAD activities (did they remind you to do them?) | *Absolutely not* | *A few* | *Neither yes nor not* | *Moderately* | *Very much* |
| 7 | Did you needed support of other persons (e.g. your son, bride/wife…) to perform HEAD activities? | *Absolutely not* | *A few* | *Neither yes nor not* | *Moderately* | *Very much* |
| 8 | When I didn’t perform activities it was because I wouldn’t | *Never* | *Rarely* | *Sometimes* | *Frequently* | *Very frequently* |
| 9 | When I didn’t perform activities it was because I couldn’t | *Never* | *Rarely* | *Sometimes* | *Frequently* | *Very frequently* |
| 10 | When I didn’t perform activities it was because the system did not work | *Never* | *Rarely* | *Sometimes* | *Frequently* | *Very frequently* |
| 11 | When I didn’t perform activities it was because even if the system worked, internet connection not | *Never* | *Rarely* | *Sometimes* | *Frequently* | *Very frequently* |
