# Supplementary material for: Efficiency and Patient-Reported Outcome Measures From Clinic to Home: The Human Empowerment Aging and Disability Program for Digital-Health Rehabilitation
Source: Front Neurol. 2019 Nov 19;10:1206. doi: 10.3389/fneur.2019.01206 (PMC6882300; doi:10.3389/fneur.2019.01206)
Supplement: Supplementary file 2 [file Table_2.docx]

**SUPPLEMENTARY MATERIALS OF THE PAPER:**

**Efficiency and Patient-Reported Outcome Measures from clinic to home: the Human Empowerment Aging and Disability program for digital-health rehabilitation**

2.0.- Table S2. Percentage of patients who followed HEAD treatment at home at least 3 times per week during 3 months of telerehabilitation. % = percentage. Adherence > 80% is reported in bold.

|  | W1 | W2 | W3 | W4 | W5 | W6 | W7 | W8 | W9 | W10 | W11 | W12 |
| --- | --- | --- | --- | --- | --- | --- | --- | --- | --- | --- | --- | --- |
| Adherence over cutoff % | | | | | | | | | | | | |
| PD | 0.67 | **1.00** | **0.89** | **1.00** | **1.00** | **1.00** | **1.00** | **0.89** | **0.89** | **0.89** | **0.89** | 0.67 |
| MS | 0.57 | **0.86** | **0.86** | **0.86** | 0.71 | 0.64 | 0.79 | 0.71 | 0.69 | 0.50 | 0.43 | 0.43 |
| Stroke | **0.92** | **0.92** | **1.00** | **0.92** | **1.00** | **0.92** | **1.00** | **0.83** | 0.75 | 0.75 | 0.67 | 0.58 |
| Whole group | 0.71 | **0.91** | **0.91** | **0.91** | **0.89** | **0.83** | **0.91** | **0.80** | 0.77 | 0.69 | 0.63 | 0.54 |
